# Promoting children’s health when a parent has a mental health problem: a mixed methods study of the experiences and views of health visitors and their co-workers

**DOI:** 10.1186/s12913-020-5015-z

**Published:** 2020-03-12

**Authors:** Louise Condon, Timothy Driscoll, Joy Merrell, Mel Storey, Amanda Thomas, Beryl Mansel, Sherrill Snelgrove

**Affiliations:** 1 College of Human and Health Sciences, Swansea University, Swansea, Wales, UK; 2 College Of Medicine, Swansea University, Swansea, Wales, UK; 3 Hywel Dda University Health Board, Carmarthen, Wales, UK

**Keywords:** Mental health, Health promotion, Child health, Mixed methods

## Abstract

**Background:**

Unrecognised and untreated parental mental illness is a major adverse childhood experience with potentially life-long consequences for health and wellbeing. In the United Kingdom (UK) health visitors provide a universal health promotion service to children aged 0–5 years, which includes safeguarding. This preventive work is highly relevant to policy aims of improving outcomes for children living with adverse childhood experiences, but is currently under researched. The aim of this study was to explore how health visitors promote young children’s wellbeing when a parent has a mental health problem, and to co-produce strategies to improve child health outcomes.

**Methods:**

A mixed methods study was conducted, consisting of a cross-sectional survey and consensus workshops in Wales, UK. In phase 1 health visitors (*n* = 174) responded to an online questionnaire designed to explore the nature and scope of their preventive work with families experiencing mental ill health. For phase 2 providers of health and other support services (*n* = 38) took part in Nominal Group Technique workshops to co-produce strategies for better joint working to protect the wellbeing of children living with parental ill health.

**Results:**

We identified that health visitors routinely provide support to families where parents have a range of mental health problems, including severe mental illness. Most practice is focused on mothers with depression, and fewer respondents were confident about working with fathers. Unmet training needs were identified in relation to adult mental illness, particularly the impact upon children. Solutions to working more effectively with professional and voluntary agencies included raising awareness of professional roles and responsibilities, timely two-way communication, taking a strengths-based approach and maintaining a focus on the child.

**Conclusions:**

This study provided evidence on the range of parental mental ill health encountered by health visitors and the strategies they use to protect children’s wellbeing. Increasing the effectiveness of joint working is key to improving outcomes for babies and young children, including greater use of voluntary sector services. This study has implications for those who commission and provide health and welfare services for children, and adult mental health services.

## Background

Mental health is a public health priority globally [1] and in the United Kingdom (UK) [2], requiring the provision of a range of primary care and secondary health services. Mental health problems range from common mental health problems, such as anxiety and depression, to severe and enduring mental illness. As around 25% of adults are likely to have a mental health problem in any year [3] many children live with parents experiencing mental health difficulties [4], and over 60% of women and 50% of men with psychiatric disorders are parents [5]. It is increasingly recognised that adverse childhood experiences (ACEs) have an impact on children’s health and wellbeing throughout the life course [6]. Parental mental ill health is a potent ACE, which reduces parents’ ability to keep their children safe from harm [7]. Parental mental illness which remains unrecognised and unaddressed contributes to an intergenerational cycle of disadvantage [8] and greater demand upon health services.

Children may cope well with parents’ short term emotional and behavioural problems but more severe and long-term issues have a significant effect upon their health [9]. Around two-thirds of children whose parents have mental illness will themselves experience mental health difficulties requiring treatment [10]. Worldwide the most common mental illness among women is depression [11], and if untreated this can create severe problems for mother, child and family [12, 13]. Babies are at greatest risk because adverse experiences, such as maternal depression, affect the optimal maturation of the child’s brain in the first 2 years of life [14], leading to poorer long-term cognitive development [15]. Long term follow-up studies show children of depressed mothers have an increased risk of mortality throughout the life-course [8].

Many parents with mental health problems provide entirely adequate care, but some children are at increased risk of harm [16]; this includes neglect, or injury when a parent is suffering from depressive mental illness or psychosis [17]. Parental mental health problems were identified as a causative factor in over 50% of serious case reviews in England [18]. Very young children are most commonly the victims of abuse, and in Wales around a third of children on the Child Protection Register are 0–4 years, with half of these under 1 year [19]. Early identification and assessment are therefore crucial to ensure babies and children living with parental mental illness are not abused or left in unsafe situations [20]. This highlights the importance of universal health services in the early years, such as those provided by health visitors [21].

UK NICE guidelines for ante and postnatal mental health [22] advocate family-inclusive practice which involves the multidisciplinary team and the voluntary sector. Such guidelines are often inconsistently applied in practice [23], and a study of one English region [24] indicated a high prevalence of parental mental illness, but inconsistent assessment of cases and provision of appropriate family support. Where parental mental illness is present it is recommended that health services provide whole-family engagement [10]. However, Ofsted [25] reported an inadequate response to parental mental illness from mental health services and a failure to adapt assessment or practice to identified family needs. Children of parents with a mental illness can be over-looked by adult-orientated services and unrecognised in child-orientated services [26].

Universal child health promotion is offered in the UK via the Healthy Child Programmes of England, Wales, Scotland and Northern Ireland [27]. These contain a range of measures to support parenting by means of planned child health promotion contacts through the early years. Health visitors are unique in the primary care team, in offering a proactive child health promotion service, which does not rely on the parent seeking help. Universal health promotion contacts offered to parents varies by country, with Scotland currently offering the most comprehensive programme in the UK with 11 home visits to all families (eight within the first year of life), compared with five in total in England. All UK countries offer more intensive child health promotion to children living in the most socio-economically deprived areas, who are assumed to have the highest health needs. In Wales the Flying Start programme provides an enhanced service to areas with high uptake of income benefits [28]. Flying Start health visitors have smaller caseloads than universal health visitors (e.g. one Flying Start health visitor per 110 pre-school children) [29], and in 2018 around 25% of children in Wales aged under 4 years received Flying Start services [30].

Research is lacking into universal parenting interventions, particularly outside the UK and Republic of Ireland [31]. Health visitors are recognised as identifying ante- and post-natal depression and delivering psychologically informed interventions [12], but beyond perinatal mental health there is little existing research. A recent review of health visiting [32] included only evidence relating to maternal depression, due to the paucity of research concerning fathers who experience mental health problems. This study will explore the extent and nature of health visitors’ support for all adult mental health, and offer solutions (produced collaboratively by the multidisciplinary team) to working more effectively to protect pre-school children’s health and wellbeing.

### Research aims

This study was designed to:

- Explore the extent and nature of health visitors’ work in maintaining pre-school children’s health and wellbeing when a parent has a mental health problem
- Identify how health visitors can work effectively with the multidisciplinary team to reduce the impact of parental mental health problems on pre-school children

## Methods

A mixed methods approach was taken in this two-phase study. Mixed methods are commonly used when taking a pragmatic approach to a research problem [33], providing a depth of knowledge that would be difficult to obtain by either method alone [34]. The two research phases (quantitative then qualitative) were not designed to replicate or triangulate each other, but to facilitate a sequential and progressive empirical investigation. This approach is designed to yield findings which are robust, fitted to local and national contexts, and with validity to allow recommendations to be made. By collecting standardised data in a systematic way [35] the survey provided evidence of current practice. Building upon survey findings, Nominal Group Technique was used to explore the research questions in greater depth [36], involving members of the multidisciplinary team in co-producing real world solutions; the main strength of this technique is consensus, rather than depth, which meets the stated aims of this study. The advantages of NGT above other consensus techniques is that the contributions of all participants are systematically sought and recorded, an important point when there are potential differences of power and status [37]. Mixed methods contributed to a complex and reflexive picture of the subject of study [38].

### Design

In phase 1 all health visitors working with families in Wales were invited to take part in an online survey, using Qualtrics software to ensure anonymity. An information sheet was devised for participants which gave details of the research question and funding, and explained that the research was being led by a University academic team. Questions were drafted by the principal investigator (LC) from knowledge of existing research plus professional experience, then discussed for content and relevance with the research team, which included a practising health visitor, a mental health nurse and a service user. The resultant questions covered the following areas: (1) number and type of parental mental health problems encountered by health visitors, (2) therapeutic strategies and interventions employed (3) views on multidisciplinary working (4) confidence and expertise in practice. As we were attempting to reach all eligible health visitors, no sample size calculation was required.

Piloting of the online questionnaire was carried out November 2017 in England with six registered heath visitors, including a safeguarding lead and an academic. Minor changes were made to data items as a result of written or verbal comments such as adding additional response options to the list of screening tools. Pilot study participants confirmed that the study was relevant to current health visiting practice, used appropriate terminology and could be completed within around 15 min. The full survey is accessible as a Supplementary file.

The sampling frame was all health visitors working in Wales, who were sent an invitation to participate in the survey via their locality managers in December 2017. Screening questions were included at the start of the questionnaire to ascertain whether participants were currently working with children and families in Wales; those who were not were excluded from analysis. Three reminders were sent to managers for distribution by email from December 2017 to February 2018. As the survey was sent via intermediaries it is not possible to ascertain whether all health visitors working in Wales (*n* = 876 full time equivalent) [39] received an invitation to participate. If participants wished to enter a prize draw they could provide their names, which were kept separate from the survey to maintain anonymity.

In phase 2 four Nominal Group Technique (NGT) workshops took place May to June 2018 in North, South and West Wales. The sample was drawn from the professional groups who had been identified in the survey as instrumental in caring for adults with mental health problems and their children; these were from health, social services and voluntary agencies. Participants were initially invited to each workshop by a link worker who had an interest in child and family mental health; names of potential link workers were provided to the research team by health visitor managers. Link workers used their local knowledge to make contact with potential participants from the range of targeted disciplines, aiming for a balance of health visitors, mental health professionals and voluntary workers. Linkworkers shared the project flyer with potential participants; if they wished to take part in the study their email address was then given to LC who provided the information sheet, gave details of the date and venue, and answered any questions. Workshops were held in venues familiar to participants, either a health centre or council offices.

Essentially the NGT is a structured group discussion during which participants generate a list of statements in response to a question, then rank the statements in order of importance [40]. Workshops were led by one researcher who had an academic and health professional background (LC or BM), assisted by two other team members (also university researchers with a health professional background) who took field notes and collated data. Team members were introduced to the group as researchers based in a University department who had a professional background in either child or mental health.

To explore the question: ‘How can health visitors work most effectively with the multidisciplinary team to promote the health of pre-school children whose parents have a mental health problem?’ the four NGT stages were followed. These are: (1) silent generation of ideas in writing; (2) round-robin feedback from group members to record each idea as a terse statement on a flip chart; (3) discussion of each recorded idea for clarification; (4) individual voting on priority ideas with the group decision being mathematically derived though rank-ordering [41, 42]. In an optional additional step, participants were given the opportunity to discuss and agree overall priorities once individual voting was completed [37]; in all workshops the top five priority interventions arising from the four-stage process were agreed as the most important to all present.

### Data analysis

Survey data was analysed using SPSS 25. A total of 189 total responses were received of which 174 were valid; 15 records were excluded from the analysis due to respondents not stating that they worked with children and families in Wales. The valid responses consisted of 14 partial and 160 complete surveys (i.e. answered the last question, even if some previous items were missing). Both partial and completed surveys were included in the analysis. We did not attempt to weight the data, account for non-response bias, or calculate a response rate, as no data were available to do this.

The views of universal health visitors (*n* = 81) and Flying Start health visitors (*n* = 77) were compared using a two-sided chi-square test at a significance level of 5%. Managers (*n* = 13) and other unspecified respondents (*n* = 3) were excluded from this comparison, as too few responded for results to be meaningful. No interactions between items were considered. Where data were available, we compared data relating to health visitors’ experiences of working with mothers versus and fathers. Responses to items asking if the health visitor had certain experiences and within what timeframe were grouped by whether the event was experienced at all or not for subgroup analysis. Missing data were logically imputed where possible, with an assumption that respondents did not do something if they did not report it. Otherwise, data were treated as missing completely at random and excluded from analysis for that question. Where respondents did not complete the survey, subsequent responses after the last completed question were not imputed, and they were treated as missing. For a question on mental health training we did not impute due to high proportions of missing data. For a question where respondents were required to rank their top five from a list of possible interventions, if more than five were ranked the subsequent ranks were disregarded in the analysis. No sensitivity analysis was undertaken.

Nominal Group Technique workshops yield quantitative and qualitative data which were analysed across groups. The challenges of collapsing, comparing and analysing data across multiple groups are well recognised [43], as differing numbers of participants and variations in statements make it difficult to compare and contrast priorities. Van Breda’s analytical method [40] gives consideration not just to the strength (sum of the votes) or relative importance of statements, but to the voting frequency, as this represents the popularity of the idea among participants. Statements generated in stage 2 which received no votes in stage 4 were excluded from the cross group quantitative analysis, but retained to add to researchers’ understanding of the ideas proposed during qualitative content analysis [40]. Remaining statements (*n* = 85) were coded into the themes and categories, with one researcher (JM) identifying preliminary themes, which were agreed by a second researcher (LC). Four researchers (JM, LC, BM and AT) then coded all statements into themes to ensure maximum rigour.

A pragmatic approach was taken to integration of data from the quantitative and qualitative elements of the study. Denscombe [33] suggests that pragmatism is the philosophical partner to mixed methods, cutting through the dualism of positivism and interpretivism. Mixed methods analyses was conducted sequentially in two phases, with the quantitative phase preceding the qualitative analysis. The initial analysis phase informed the subsequent phase, which built upon these findings. Following the analysis of quantitative and qualitative data from NGT workshops, findings were collated and synthesised with survey findings. At this stage the data were fully integrated and interpreted as coherent whole.

### Findings

#### Survey (phase 1) findings

Virtually all respondents (99%, 172/174) stated they had worked with a parent who had a mental health problem. Respondents were universal health visitors (47%, 81/174) and Flying Start health visitors (44%, 77/174), with 9% (16/174) managers or missing (see Table 1). Findings are presented as follows: (1) the type of parental mental health problems encountered by health visitors in their practice, (2) the wellbeing of children, (3) strategies and interventions employed by health visitors, and (4) training.

**Table 1:** Details of participants (and number of statements generated in each workshop)

Workshop	Number of Participants	Participants’ roles	(Number of statements Generated in each workshop)
PHASE 1			
Survey	174	81 x health visitors 77 x Flying Start health visitors 10 x health visitor managers^a^ 3 x Flying Start managers^a^ 2 x Others^a^ (1 “team leader” and 1 unspecified). 1 x Unknown^a^	N/A
PHASE 2			
Workshop 1	15	6 x health visitors 3 x community psychiatric nurses 3 x early years liaison workers 2 x community nursery nurses 1 x third sector worker	30
Workshop 2	4	3 x health visitors 1 x community nursery nurses	27
Workshop 3	12	8 x health visitors 2 x community nursery nurses 1 x third sector worker 1 x social worker	32
Workshop 4	7	5 x health visitors 1 x third sector worker 1 x midwife	30
Workshop Total	**38**	**22 health visitors** **16 other service providers**	**119**

^a^(these groups were treated as missing for analyses by arm)

##### Parental mental health problems encountered by health visiting services

The most common maternal mental health problems were anxiety and depression, which is unsurprising as these are the most common within the general population in the UK [2] and globally [11]. Post-natal depression was highly prevalent (95% of respondents had worked with a mother experiencing this), and ante-natal depression also commonly encountered (see Table 2). Depression was the most commonly perceived mental health problem of fathers, followed by substance misuse disorder. Severe mental illness was encountered in both parents, including psychosis, schizoaffective disorder, bipolar disorder, attention-deficit/hyperactivity disorder (ADHD) and autism spectrum disorders. Severe mental illness was encountered more frequently in mothers than fathers, apart from ADHD and autism spectrum disorders (see Table 2).

**Table 2:** Number and type of parental mental health problems encountered by universal (HV) and Flying Start (FS) health visitors in their practice

	Mother				Father				Mother vs father
	HV *n* = 78 missing = 3	FS *n* = 73 missing = 4	All health visitors *n* = 164 missing = 10	*p*	HV *n* = 79 missing = 2	FS *n* = 70 missing = 7	All health visitors *n* = 163 missing = 11	*p*	*p*
Anxiety	96% (*n* = 75)	99% (*n* = 72)	96% (*n* = 158)	0.541	76% (*n* = 60)	81% (*n* = 57)	78% (*n* = 125)	0.692	< 0.001^a^
Post-natal depression	96% (*n* = 75/78)	95% (*n* = 69)	95% (*n* = 156)	0.416	**–**	**–**	–	–	–
Depression	94% (*n* = 73)	96% (*n* = 70)	93% (*n* = 153)	0.594	91% (*n* = 72)	90% (*n* = 63)	89% (*n* = 144)	0.301	0.454
Panic attacks	77% (*n* = 60)	84% (*n* = 61)	80% (*n* = 131)	0.426	35% (*n* = 28)	41% (*n* = 29)	39% (*n* = 62)	0.706	< 0.001^a^
Substance misuse disorder (drugs)	73% (*n* = 57)	82% (n = 60)	77% (*n* = 127)	0.301	84% (*n* = 66)	83% (*n* = 58)	83% (*n* = 134)	0.783	0.169
Ante natal depression	72% (*n* = 56/78)	77% (n = 56)	74% (*n* = 121)	0.416	**–**	**–**	–	–	–
Bipolar disorder	69% (*n* = 54)	73% (*n* = 53)	71% (*n* = 117)	0.590	46% (*n* = 36)	39% (*n* = 27)	42% (*n* = 68)	0.647	< 0.001^a^
Suicidal thoughts	67% (*n* = 52)	70% (*n* = 51)	68% (n = 112)	0.908	41% (*n* = 32)	37% (*n* = 26)	40% (*n* = 64)	0.825	< 0.001^a^
Alcohol use disorder	67% (*n* = 52)	68% (*n* = 50)	67% (*n* = 110)	0.237	72% (n = 57)	74% (n = 52)	74% (*n* = 119)	0.931	0.217
Obsessive–compulsive disorder	65% (*n* = 51)	58% (*n* = 42)	62% (*n* = 101)	0.100	28% (*n* = 22)	27% (n = 19)	27% (*n* = 43)	0.724	< 0.001^a^
Post-traumatic stress disorder	55% (*n* = 43)	60% (*n* = 44)	57% (*n* = 94)	0.573	54% (*n* = 43)	47% (*n* = 33)	52% (*n* = 83)	0.398	0.383
Eating disorder	55% *n* = 43)	58% (*n* = 42)	56% (*n* = 92)	0.634	9% (*n* = 7)	7% (*n* = 5)	8% (*n* = 13)	0.895	< 0.001^a^
Psychosis	44% (*n* = 34)	45% (*n* = 33)	46% (*n* = 76)	0.619	10% (*n* = 8)	21% (*n* = 15)	16% (*n* = 26)	0.124	< 0.001^a^
Attention deficit hyperactivity disorder	33% (*n* = 26)	47% (*n* = 34)	38% (*n* = 62)	0.192	51% (*n* = 40)	54% (*n* = 38)	52% (*n* = 84)	0.523	0.013^a^
Autistic spectrum disorders	28% (*n* = 22)	33% (*n* = 24)	29% (*n* = 48)	0.712	42% (*n* = 33)	34% (*n* = 24)	38% (*n* = 31)	0.544	0.063
Schizoaffective disorder	23% (*n* = 18)	23% (*n* = 17)	24% (*n* = 39)	0.889	24% (*n* = 19)	29% (*n* = 20)	27% (*n* = 43)	0.777	0.295

^a^Result is statistically significant at the 5% significance level

NB Numbers of Health Visitors and Flying Start Health Visitors may not sum to the total (all health visitors) due to responses from managers being excluded from subgroup analysis

There were statistically significant differences by gender in types of mental health problems identified, with all heath visitors observing mothers with more panic attacks, suicidal thoughts, obsessive compulsive disorder and eating disorders. Fathers were more likely to be known to have autistic spectrum disorder, which accords with prevalence within the general population and ADHD, where rates are roughly comparable for men and women [44]. Numbers of fathers identified with substance use and alcohol use disorders were higher than in mothers but there was no statistically significant difference (see Table 2). On no measure were significant differences found between the responses of universal and Flying Start health visitors, indicating that both encountered a similar range of mental health problems among the families with whom they work, despite Flying Start being targeted to areas of deprivation.

##### The impact upon children of parents’ mental ill health

A number of adverse childhood experiences (ACEs) accompanied parental mental health problems. Respondents had witnessed co-occurring domestic abuse (91%, 145/160), breakdown of the parents’ relationship (88%, 141/160), social isolation of the family (84%, 135/160), parents not being able to afford essentials for children (79%, 127/160), and parental incarceration (65%, 145/160). This indicates the high health and social needs of the pre-school children with whom all health visitors work. In children respondents had observed the following difficulties: developmental (74%, 119/160), behavioural (71%, 113/160) and attachment (58%, 92/160). Where a parent had a mental problem most respondents had held responsibility for children ‘*in need of care and support’* (89%, 143/160) and ‘*at risk of harm’* (84% (135/160).

Overall, respondents saw the value of their role, with 84% (133/158) agreeing that their work helped to keep children safe. The top five ways in which universal (HVs) and Flying Start health visitors (FS) keep children safe were ranked from an 11 item list:

1. Developing a relationship of trust with the family **(ranked 1st by FS, 2nd by HVs,*p*** **= 0.207)**
2. Focusing on the wellbeing and safety of the child **(ranked 1st by HVs, 2nd by FS,*p*** **= 0.0.203)**
3. Offering a targeted service when parents need more help **(ranked 3rd by both HVs and FS,*p*** **= 0.293)**
4. Knowing the family well **(ranked 4th by both HVs and FS,*p*** **= 0.804)**
5. Working well with children’s safeguarding agencies **(ranked 5th by both HVs and FS,*p*** **= 0.661)**

A weighted average of scores was used to determine rankings for the whole sample, and comparison made between groups. The Mann-Whitney U-test showed a significant difference in rank between universal and Flying Start respondents for “offering a universal service to all families”, which was ranked joint 6th by HVs and 9th by FS (*p* = 0.022). No other significant differences were found, which demonstrated the value placed by both universal and targeted services on family relationships and the primary importance of the child’s wellbeing.

##### Strategies and interventions employed by health visitors

Most respondents were confident in what actions to take when a parent had a mental health problem (80%, 125/157), and aware of organisations to which they could refer parents (73%, 114/157). Confidence in supporting a mother with a mental health problem was relativity high (73%; *n* = 115/158), with far fewer confident in supporting a father (40% (*n* = 63/158); there was a significant difference of *p* < 0.001. Mental health assessment tools were most commonly administered to mothers. The Edinburgh Postnatal Depression Score (EPDS) was used by 89% (143/160), with far fewer (40%; *n* = 64/160) using the Generalized Anxiety and Depression Score (GADS). The Family Resilience Assessment Instrument and Tool (FRAIT) had been administered to mothers by 79% of respondents (*n* = 127/160). FRAIT had been administered to fathers by 51% of respondents (82/160), EPDS to fathers by 7% (11/160) and GADS to fathers by 2% (3/160). Once a mental health problem was identified, respondents were asked to indicate which strategies and interventions they used to support mothers from a list provided (see Table 3). Only 6% (10/160) indicated they would use strategies and interventions other than those provided in the questionnaire, these were referral to another member of the health visiting team.

**Table 3:** Strategies and therapeutic interventions offered by universal (HV) and Flying Start (FS) health visitors plus referral to other agencies

	HV	FS	Overall	*p*-value
Strategies suggested to mother				
1. Attend Flying Start (if eligible)	25% (*n* = 19/77)	85% (*n* = 60/71)	54% (*n* = 87/160)	< 0.001
2. Attend a peer support group (not HV led)	43% (*n* = 33/77)	55% (*n* = 39/71)	48% (*n* = 77/160)	0.142
3. Access facilitated self-help	31% (*n* = 24/77)	35% (*n* = 25/71)	36% (*n* = 57/160)	0.602
4. Attend a peer support group (HV led)	23% (*n* = 18/77)	28% (*n* = 20/71)	29% (*n* = 46/160)	0.505
5. Contact a peer-led telephone support service	6% (*n* = 5/77)	8% (*n* = 6/71)	8% (*n* = 13/160)	0.650
Intervention by health visitor				
1. Offer more one to one contacts	97% (*n* = 75/77)	99% (*n* = 70/71)	98% (*n* = 157/160)	0.608
2. Offer a package of listening visits/non-directive counselling explain in discussion	84% (*n* = 65/77)	90% (*n* = 64/71)	87% (*n* = 139/160)	0.298
3. Offer MHFA (mental health first aid) support	12% (*n* = 9/77)	7% (*n* = 5/71)	11% (*n* = 18/160)	0.335
Referral to other agencies				
1. Refer to the GP	95% (*n* = 73/77)	99% (*n* = 70/71)	97% (*n* = 155/160)	0.203
2. Refer to perinatal mental health services	82% (*n* = 63/77)	79% (*n* = 56/71)	81% (*n* = 129/160)	0.652
3. Refer to mental health services	56% (*n* = 43/77)	68% (*n* = 48/71)	62% (*n* = 99/160)	0.142
4. Refer to voluntary agencies/third sector services	42% (*n* = 32/77)	31% (*n* = 22/71)	36% (58/160)	0.182
5. Refer to family intervention services, e.g. family therapy	25% (*n* = 19/77)	21% (*n* = 15/71)	24% (39/160)	0.608

The most common self-help strategy suggested to mothers was attending a peer support group, usually a group run by a non-health visitor agency. Accessing facilitated self-help was a strategy suggested by less than a third of health visitors overall, and only 8% (*n* = 13/160; missing = 14) suggested mothers contact a peer-led telephone support service. Instead the therapeutic interventions most commonly offered were those provided by health visitors on a one-to-one basis, in the home. Almost all health visitors would offer more contacts, with 87% (*n* = 139/160; missing = 14) offering listening visits.

Referrals to other agencies are best understood in the context of respondents’ agreement with Likert statements (see Table 4), which were designed to offer greater insight into health visitors’ views on working with other services. The high numbers of GP referrals may be linked to the high proportion who stated that referrals to mental health services were made via the GP (70%) (*n* = 105/150; missing = 24). Guidelines for referring to mental health services were found clear by less than half of respondents. Referrals to perinatal mental health services were common, but only 42% (*n* = 66/157; missing = 17) of respondents found these easy to access. Around three quarters of respondents agreed that thresholds for mental health and safeguarding services were very high. A consequence of this was that 65% (*n* = 103/158; missing = 16) of respondents agreed that as health visiting is a universal service, health visitors are often alone in supporting families. Almost half of respondents considered that the voluntary sector contributes to keeping children safe. There was no significant difference between the responses of generic and Flying Start health visitors in relation to referral strategies or agreement with Likert statements.

**Table 4:** Likert statements on universal (HV) and Flying Start (FS) health visitors’ referrals to other agencies

In my area	Strongly agree/Agree (HV)	Strongly agree/Agree (FS)	Strongly agree/Agree (overall)	*P*-value
Thresholds for children’s safeguarding services are very high	75% (58/77)	75% (49/65)	76% (117/154)	0.912
Thresholds for mental health services are very high	73% (56/77)	72% (47/65)	73% (112/154)	0.435
HVs refer clients for mental health services via the GP	68% (*n* = 50/74)	74% (*n* = 48/65)	70% (*n* = 105/150)	0.141
As Health visiting is a universal service HVs are often alone in supporting families	66% (51/77)	62% (43/69)	65% (*n* = 103/158)	0.622
Third sector services contribute to keeping children safe	51% (*n* = 38/74)	45% (*n* = 30/65)	48% (*n* = 73/153)	0.393
There are clear guidelines for referring to mental health services	43% (*n* = 33/76)	43% (*n* = 29/68)	42% (*n* = 66/156)	0.494
Perinatal mental health services are easy to access	38% (*n* = 29/76)	48% (*n* = 33/69)	42% (*n* = 66/157)	0.453

##### Training

Demand for additional mental health training was high with 91% (*n* = 142/156) requesting more training on how to work effectively with parents, 95% (*n* = 148/156) on working with fathers, and 94% (*n* = 146/156) requested interdisciplinary training. Mental health training was commonly received during the initial health visiting course (76%; *n* = 112/148). 84% (*n* = 80/95) received some training post-qualification, but a high proportion of missing responses (79/174) mean this should be interpreted with care. A few respondents (*n* = 5) stated they were trained mental health nurses.

#### Consensus workshops (phase 2) findings

Nominal Group technique workshops were made up of health visitors and service providers from health, social care and voluntary sectors. The majority of those who had expressed an interest in the project via the link worker, and received an information sheet from LC attended; those who did not attend gave reasons such as sickness, either of themselves or their child. These were identified as key members of the multidisciplinary team from survey findings; all invited groups were represented by at least one worker in one group, with the exception of GPs. Numbers of participants varied (4–15), with an average of eight. Workshops lasted between 2.5 and 3 h. Table 1 contains details of participants’ professional roles. Irrespective of the size of the group the number of statements generated during the 5 stage NGT process approximated 30 (see Table 1). At the start of each workshop participants were briefly informed of the survey findings (i.e. the contents of Tables 1, 2, 3 and 4).

Findings are presented in Table 5, with the full 85 statements being reported in the supplementary materials online. Six overarching themes were identified and reported below. In addition to referring to the priority statements, discussion within workshops is reported where this contributes to understanding of the theme. The theme of ‘Referrals’ was analysed quantitatively as a separate theme, however, it is discussed below as a co-theme to service provision as workshop participants identified these topics as closely interrelated.

**Table 5:** The Top Five Prioritised statements within each theme (based on average score)

Theme	Group	Statements	Scoring	Total	Average	Voted for as a priority?
Services	2	Provide support groups for early intervention	5,4,4	13	3.25	yes
	2	Employ a PMH (perinatal mental health) specialist with no caseload	3,4,3,2	12	3.00	yes
	3	Health visitors have direct access to tangible support	4,1,3,3,5,2	18	1.50	yes
	2	Joint working to bring together all specialities	5	5	1.25	yes
	2	Improved awareness of the services available among multidisciplinary team (MDT)	5	5	1.25	yes
Communication	1	Improve communication pathways in MDT	4,1,5,5,5,1,5,5,5,1,5,1	43	2.96	yes
	4	Talk to parents routinely about mental health	5,3,3	11	1.57	yes
	4	Ensure the client’s voice is heard when making assessments	4,4	8	1.14	yes
	2	Listen to the family rather than giving out information	4	4	1.00	no
	3	Effective discussion with MDT and family	5,4	9	0.75	no
Education and Training	2	Better understanding of mental health among health visitor team	3,2,2,5	12	3.00	yes
	4	Better training in psychological support to improve interventions	4,5,5,2	16	2.29	yes
	1	Education in mental health for health visitor staff	1,3,2,3,3,4,4,3,2	25	1.72	yes
	3	Health visitors need training in understanding mental health issues	3,5,4	12	1.00	yes
	4	Mental Health First Aid Training (MHFA) needed	3,4	7	1.00	yes
Focus on Strengths	3	Build a trusting relationship	5,2,4,4,1	16	1.33	yes
	1	Discuss safety plans with the family	5,3,5,5	18	1.24	yes
	3	Work with MDT on signs of safety, setting sustainable goals	5,2,3,2	12	1.00	yes
	4	Support families to overcome barriers to accessing additional support	3,3	6	0.86	no
	1	Build a ‘team around the health visitor’	1,4,4	9	0.62	no
Focus on child	4	Keep focus on child when supporting parental mental health issues	5,5,5,5	20	2.86	yes
	3	Maintain a focus on child wellbeing within MDT	2,5,1,3,5,1	17	1.42	yes
	1	Wider definition of risk to include neurodevelopmental risk	5,5,3,5	18	1.24	yes
	3	Recognise health visitors’ unique ability to assess home environment	4,5	9	0.75	no
	4	Develop health visitors” ability to recognise impact upon child	4	4	0.57	no
Referrals	2	Ensure timely and appropriate referrals to other services	3	3	0.75	no
	4	Make clear referral pathways	4,1	5	0.71	no
	1	Ensure appropriate referrals with clear roles and responsibilities	5,4,1	10	0.69	yes
	3	Health visitors understand roles and pathways of MDT	4,3	7	0.58	no
	4	Health visitors can refer directly to mental health services	1	1	0.14	no

##### Services and referrals

In line with survey findings, services provided by health visitors were rated as the highest priority, specifically support groups and a specialist mental health role. Health visitors saw a need for direct, tangible support to enable parents to attend to their mental health needs, for instance by providing funding for childcare or transport costs. Obstacles to parents attending appointments with services outside the home were perceived as: distance to travel, lack of child care or fear of leaving the house. Voluntary sector workers made a strong case for using their services to provide necessary support, for instance by befriending clients, accompanying them to appointments and offering parenting support at home. Participants identified that the multidisciplinary team as a whole lack awareness of the services each professional or voluntary agency provides. An example of better working together was health visitors contributing to perinatal mental health team discussions in pregnancy.

Referral was a specific area of concern for participants, with recommendations on how to ensure they are timely and made to the appropriate agency. Parity of referral was seen as a problem as more services are often available to Flying Start health visitors, for instance mental health workers as part of the team. Referral pathways were described as unclear by all health visitors and mental health workers. A major barrier to accessing local primary mental health services for health visitors was being required to refer parents via the GP. One health visitor described the perversity of this, given her detailed knowledge of the family circumstances and the home-environment, compared with a time limited, practice-based GP appointment. Secondary service providers suggested that health visitors routinely provide explicit information about the child and family circumstances, for the referral agency to understand the level of need and urgency. When referrals were not accepted, health visitors requested feedback in order to understand the reasons why, and also guidance on alternative sources of support for adult mental health issues.

##### Communication

Regular liaison and more informal contacts which allowed for informal sharing outside statutory meetings were suggested to improve communication, with GPs a specific priority. A way of improving knowledge of other agencies’ roles would be meetings with the multidisciplinary team as induction to a new post. Feedback within the team should be two-way, as health visitor participants reported experiences of sharing information which were not reciprocated. This was important when referrals were not accepted, or clients did not attend appointments and were therefore discharged, as this had implications for dependent children. Health visitors stated that they retained responsibility for the child’s wellbeing, irrespective of whether the parent was offered, accepted or attended appropriate treatment. Communication with parents could be improved by health visitors routinely talking about mental health, not just at statutory child health promotion contacts; this would assist with prevention and early identification of mental health issues. Emphasis was put on working in partnership with clients, for instance active listening and ensuring that clients’ voices are heard when making assessments.

##### Education and training

This theme was ranked as a top five priority in all groups, the only theme to have this level of unanimous support. Prioritised solutions were more consistent and less complex than for the preceding themes. It was agreed that more education in adult mental health is needed for health visitors and their teams. An interdisciplinary training package was requested, such as Mental Health First Aid, as this would contribute to workers understanding each other’s roles, and a common body of knowledge. Training in psychological support would improve the interventions health visitors could offer to families. Once trained, clinical supervision of a reflective nature would assist health visitors in keeping up to date in adult mental health, as would carrying out joint visits to clients with mental health practitioners.

##### Focus on strengths

The strengths-based approach was most familiar to participants who had worked outside mainstream health visiting, for instance in a substance misuse team. This approach builds upon the strengths and resources within the family, aiming to build resilience and promote positive mental health rather than focusing on deficiencies. For instance, a health visitor could meet a mother experiencing depression and her family to plan for ‘a bad day’; support could then be identified from both family and professionals. Participants highlighted the heightened risk for the child whose mother was experiencing acute mental health difficulties in an unsupportive or chaotic environment, compared with a child living within a family which was otherwise well functioning. Safety plans for the children could be discussed with the extended family and the multidisciplinary team, identifying ‘*signs of safety’* and setting ‘*sustainable goals’*. In order to work effectively and acceptably with families, particularly fathers, the importance of using positive language was emphasised.

In order to maintain health visitors’ strength and resilience some early years support workers suggested that a ‘*team around the health visitor’* was needed. Such workers described their own caseloads as being reduced when demand was high, and they had regular supervision from a psychologist. In contrast they perceived health visitors as being consistently overloaded, without the supervision required to build practitioners’ resilience, and isolated in their continued responsibility for child wellbeing.

##### Focus on the child

Health visitors in all workshops considered that maintaining a focus on the child was one of their prime strengths. On occasion the multidisciplinary team was perceived as focusing on the adults’ mental health without sufficiently taking into account the wellbeing and safety of the dependent child. Health visitors were aided in focusing on the child by their familiarity with the home environment, and expertise in assessing parent/child attachment. Assessments of risk should include neurodevelopmental needs, in addition to highly visible factors such as whether a child is dirty. A wider definition of risk would recognise the importance of early attachment for brain development, and its subsequent impact upon children’s health and future life chances. Heath visitors were described as being able to make in-depth, home based assessments of attachment but these were not sufficiently recognised or taken into account by the wider multidisciplinary team. However, in order to perform this child advocacy role more effectively health visitors needed greater understanding of severe mental health issues, in particular the impact upon the pre-school child.

## Discussion

Living with a parental mental health problem is a common adverse childhood experience [45], with implications for the psycho-social health of future generations. As providers of universal health promotion services health visitors have a unique role in protecting and maintaining children’s health and wellbeing in adverse circumstances. This study fulfilled the study aims of (1) exploring health visitors’ work in these specific circumstances, and (2) identifying practice based solutions. It showed health visitors offer services to families who have a range of mental health problems, ranging from anxiety and depression to serious mental illness. Importantly Flying Start and universal health visitors reported no difference in the problems observed. The number of problems experienced may differ between universal and intensively targeted services (this survey was not designed to detect this), but the range and severity of mental health problems did not differ. This research therefore provides new evidence with implications for commissioners and providers of services. Whereas existing research has focused on perinatal mental health to the exclusion of other mental health disorders, this study has explored both mothers and fathers’ mental health needs in the pre-school period. Despite the prevalence of mental ill health, and the consequent impact upon children, this area of health visitors’ work with both sexes has been unaccountably neglected in research and policy.

Fathers are recognised as significant protective or risk factors in vulnerable children’s lives, and recent child development research indicates that fathers influence their children independently of mothers and equally strongly [46]. As 10% of new fathers worldwide are estimated to experience postnatal depression [47] and in a large UK survey over a third of first-time fathers expressed concerns about their mental health [48], it is apparent that both parents merit preventive interventions. In a landmark step the recent NHS Long Term Plan for England [49] proposes mental health assessment for the partners of postnatally depressed mothers in England, which is a step towards addressing the mental health needs of partners. This study indicated that such a step would be of benefit in Wales to address fathers’ needs and better protect their children.

This study demonstrated that health visitors are confident in the core skills of home visiting, assessment, developing trusting relationships and offering one-to-one support specifically to mothers, including therapeutic interventions. This supports the findings of existing research [50–52]. However, our findings also suggest that this focus on health-visitor led interventions may be to the exclusion of self-help options and the use of voluntary sector services, which are now widely promoted in primary care [53]. Participants expressed a need for greater knowledge of adult mental illness, including fathers, by means of interdisciplinary training. The need for such training has previously been recognised in policy and in empirical research [54–56]. Interdisciplinary training is required to ensure a shared understanding of mental health and illness (mild-moderate and severe) and the increase awareness and understanding of complementary roles.

Health visitors’ easy access to children and their families is based on the universal contacts of Healthy Child Programmes, which facilitate early intervention and safeguarding [57]. These programmes are subject to change in the light of new research and knowledge, as well as shifting political ideologies and levels of investment [58–60]. Currently there is divergence in the scope and intensity of the child health programmes of the four UK countries, despite all sharing the same clinical evidence base [61–63]. There is also concern about the extent to which child health programmes defined in policy are delivered in practice. Welsh statistics show variation in contacts delivered, for instance 91% of eligible children receive a timely new birth contact but only 53% at 3.5 years [30], which suggests prioritisation by practitioners. However, adding further mandated assessments risks overstretching budgets and increasing caseload numbers to unsustainable levels, if no additional resource is provided [28]. Heavy workloads and time pressures are identified barriers to optimal perinatal mental health service delivery [56], and policies to ensure adequate nurse staffing in public health nursing are long overdue [64].

This study also produced recommendations for practice (second research aim) which were produced collaboratively by the multidisciplinary team. The widest interpretation of the team was taken, including all those involved with supporting children, such as the voluntary sector. This is wide-ringing partnership approach is supported by the recent policy initiative in England (‘A Better Start’) which in one trial area was led by a children’s charity who commissioned more health visitor contacts to improve child development [65]. In our study representatives from the multi-disciplinary team reached a consensus on three areas where service and attitudinal changes could productively be made in order to improve outcomes. These are: more effective joint working, working more positively with parents and focusing on the child. All areas have implications for the policy making and service delivery.

Firstly, more frequent and effective interdisciplinary working is a pre-requisite for improved communication. Better liaison, particularly between health visitors and GPs, would improve parents’ access to primary mental health services. Local primary mental health services in Wales have been the subject of policy change to facilitate timely access to early support [55], but this study shows barriers still exist, primarily at the level of GP referral. At a UK policy level, health visitors’ adult mental health role may be insufficiently recognised; this is suggested by ‘listening visits’ to mothers being omitted from perinatal guidelines [66], and in Wales health visitors being recognised as tier 1 practitioners (frontline service providers who recognise, assess and intervene with mental health problems) for children, but not for adults [55]. Establishing relationships between primary and secondary care professionals is a route to integrating services, in order to promote shared norms and to improve cultural understanding of service thresholds and their ability to work together [67].

Secondly, a strengths-based approach was identified as the most effective way of working with parents. This has begun in the use of the Family Resilience Assessment Instrument (FRAIT) tool [68] and in seeking ‘signs of safety’ [69], but needs further development for instance in focused inclusion of fathers. A review of child welfare literature [46] highlights that children are placed at increased risk if potentially dangerous fathers are not engaged, and are also significantly disadvantaged if supportive fathers are not engaged. This study suggests specific techniques to engage fathers, such as using consistently positive language. A strengths-based approach builds upon health visitors’ existing skills in relationship building and their privileged position as a universal visitor to families [70]. Working therapeutically with fathers raises training, capacity and capability issues for health visiting services. However, this research has demonstrated that health visitors work routinely with families where parents have a range of mental health problems, to promote the health of the child by working therapeutically with parents. Health visitors’ universal role, home visiting and ongoing responsibility for the child ensures they continue to provide a service for families, even when parents are not eligible for, do not attend or reject mental health services.

Thirdly, health visitors considered they have a lead role in keeping the focus on the child, and hence reducing harm; however this was not always recognised or sufficiently valued by the multidisciplinary team. In the first 1000 days, a crucial period for attachment and baby brain development, health visitors may be only professionals with routine access to the child. Health visitors are well placed to assess attachment, and many participants in this study stated they were skilled in doing this. A caveat is that previous research has identified a skills gap in assessment of mother-infant interactions [71, 72], and more research is required about how health visitors support the establishment of parent-child relationships [73]. Health visitors are lead safeguarding practitioners in primary care, holding generic expertise in health promotion and safeguarding. A contributory factor in serious case reviews is professionals taking too narrow a view of their responsibility, seeing it solely from the perspective of their own discipline [18]. This study suggests that health visitors can be isolated in taking responsibility for the child’s safety when a parent has an untreated mental health problem.

### Limitations

The extent to which survey participants are representative of all health visitors practising in Wales or the UK is not known, and the number of responses is small in relation to the total number of health visitors in Wales. Relying on managers to distribute the survey may have increased recruitment bias, as we cannot be certain that all potential participants received the survey. A relatively large number of survey items compared to the number of responses means that multiplicity of analysis is a concern. However, the majority of statistically significant findings had very small *p*-values (p < < 0.05), and so would remain significant at a much higher significance level. Proportionately higher numbers of Flying Start health visitors responded to the survey, who works intensively with smaller caseloads and additional resources. This increases the likelihood that the findings from this study are more positive than if the study was conducted in a country with a less comprehensive Healthy Child Programme, and fewer intensive services for areas of deprivation. It was not possible to recruit GPs to any Nominal Group Technique workshop, which meant their views were unrepresented. Epidemiological data on the incidence of parental mental health problems and the service provided by health visitors would be required to gain a fuller picture, in addition to exploration of service users’ perceptions. Study findings are applicable to the UK context, and offer insights relevant to public health nurses who provide child health promotion elsewhere [74]. In countries dominated by private healthcare provision developmental reviews are generally offered by doctors [75], and the applicability of these findings is therefore reduced.

## Conclusion

More focus is required on the therapeutic mental health work of health visitors, in policy and practice, and from within and outside the profession. Implications for service providers, educators and commissioners are widespread. In order to work more effectively, health visitors require more in-depth knowledge of adult mental illness as this is a potent factor affecting children’s physical, social and emotional development. More effective use could be made by health visitors of self-support and the voluntary sector. While retaining a focus on the child, a whole family approach is recommended which builds on family and community strengths and is inclusive of fathers. As a universal, non-stigmatising service, health visitors are able to work with both parents to address mental health issues and improve parenting capacity. This study indicates how this potential can be maximised to improve outcomes for children.

## Supplementary information

**Additional file 1.** MANIFeST (Maintaining child and family safety when a parent has a mental health problem).

**Additional file 2.**

## Data Availability

In addition to data presented in this manuscript the full Nominal Group Technique workshop findings are presented in a supplementary file.

## Abbreviations

ACE: Adverse childhood experience
ADHD: Attention-deficit/hyperactivity disorder
EPDS: Edinburgh postnatal depression scale
FRAIT: Family resilience and assessment instrument and tool
FS: Flying Start
GADs: Generalised anxiety and depression scale
GP: General practitioner
MDT: Multidisciplinary team
MHFA: Mental health first aid
NGT: Nominal group technique
PMH: Perinatal mental health
UK: United Kingdom
