# Supplementary material for: Promoting children’s health when a parent has a mental health problem: a mixed methods study of the experiences and views of health visitors and their co-workers
Source: BMC Health Serv Res. 2020 Mar 12;20:195. doi: 10.1186/s12913-020-5015-z (PMC7068942; doi:10.1186/s12913-020-5015-z)
Supplement: Supplementary file 1 — Additional file 1. MANIFeST (Maintaining child and family safety when a parent has a mental health problem). [file 12913_2020_5015_MOESM1_ESM.pdf]

MANIFeST (Maintaining child and family safety when a parent has a mental health problem)

Start of Block: Block 1

Information
This survey will provide important information about the health visitor's role and provide evidence on current practice and the context in which it takes place. Please read the information below for full information about the research study and how the findings will be used.

If taking the survey on mobile phone or tablet, you may need to look at the bottom of the screen for the continuation bar.

Study title
MANIFeST- Maintaining child and family safety when a parent has a mental health problem: a nurse-led participatory project

What is the purpose of the study?
This study aims to explore health visitors' work in maintaining child and family safety when the parent of a pre-school child has a mental health problem. The study has two parts:

Phase 1: An online survey will be distributed to all health visitors working with children aged 0-5 years in Wales

Phase 2: Participatory workshops will be carried out with health visitors and key stakeholders in four geographically diverse locations in Wales.

You are being invited to take part in phase 1 which is an online survey to ask about your work. This will help us understand the extent and nature of health visitors' work and find out about training and any interventions you are involved in.

Why have I been chosen?
You have been chosen because you are a registered health visitor who works in Wales. All health visitors in Wales, whether in practitioner or managerial roles, are invited to take part.

Do I have to take part?
No, it is up to you to decide whether or not to take part. If you do take part, you simply complete the questions.

What will happen to me if I take part?
In this online survey there are questions about your work with families when a parent has a mental health problem, including how you keep children safe.

What do I have to do?
If you decide to participate you just need to start the survey using the link provided.

What are the disadvantages and risks of taking part?
It will take about 15 minutes to complete the online questionnaire, so you will need to find time to take part. It is up to you whether you complete the survey at home or at work. NHS ethical guidelines say that you are allowed to participate in research at work if this does not take up too much time and it does not impact on your other work. We have designed the survey to take about 15 minutes but some people may take less or more time.

What are the possible benefits of taking part?
The study is designed to produce evidence on the contribution of health visitors to keeping children safe when a parent has a mental health problem. No such large scale work has previously been done on this important area of public health and the findings will therefore be of use throughout the UK and beyond. This is your opportunity to tell us about your important work.

Will my taking part in the study be kept confidential?
Yes. All the information about your participation in this study will be kept strictly confidential. If you make free text comments in the online survey these may be used in future publications and public reports from the study. All quotations used will be anonymised to maintain the anonymity of participants.

All information which is collected during the course of the study will be kept strictly confidential. Online data will be securely stored in the College of Human and Health Science. At the end of the study all data will be stored securely for 5 years, before being destroyed

What will happen if I don’t want to carry on with the study?
If you start the online survey you are free to complete as many or as few questions as you wish. You can withdraw at any time whilst completing the questionnaire without giving a reason why. Once you have submitted the completed survey it is not possible to withdraw your individual responses.

Who is organising and funding the research?
The research project is being organised by Swansea University, and funded by the General Nursing Council Trust.

Who has reviewed the study?
This study has been reviewed and approved by the College of Human and Health Sciences Research and Ethics Committee.

What if there is a problem?
If you have a problem about any aspect of this study please contact Professor Louise Condon who is the project lead at L.J.Condon@Swansea.ac.uk or on 01792 295643. If you remain unhappy after talking to Louise you can contact a member of the College's senior management team. Details are available from the College website or from College of Human and Health Sciences, Swansea University, Swansea SA2 8PP. Telephone: 01792 513801.

This study seeks to explore health visitors' work with women and men who are parents. Our definition of parents includes adoptive parents, step-parents and partners who have a parenting role**.**

End of Block: Block 1

Start of Block: Default Question Block

Q1 Do you work with pre-school children and their families in Wales in your current post (either as a practitioner or manager)?

- ▢ Yes (1)
- ▢ No (2)

Skip To: Q15 If Do you work with pre-school children and their families in Wales in your current post (either as... = No

Q2 2 If yes, which of the following best describes your current post? (Please tick one box only)

- ○ Health visitor for children and families (1)
- ○ Flying Start health visitor (2)
- ○ Health visitor manager (3)
- ○ Flying Start manager (4)
- ○ Other NHS manager (5)
- ○ Other, please describe (6) ____

Q3 Have you worked as a health visitor with families where a parent has a mental health problem?

- ○ Yes (1)
- ○ No (2)

Skip To: Q15 If Have you worked as a health visitor with families where a parent has a mental health problem? = No

---

Page Break

Q4 If yes, please tick the mental health problems which a **mother** you have worked with has experienced.

	Yes (1)	No (2)	Don't know (3)
Ante natal depression (1)	○	○	○
Post natal depression (2)	○	○	○
Depression (3)	○	○	○
Anxiety (4)	○	○	○
Panic attacks (5)	○	○	○
Psychosis (6)	○	○	○
Borderline personality disorder (7)	○	○	○
Bipolar disorder (8)	○	○	○
Schizoaffective disorder (9)	○	○	○
Obsessive–compulsive disorder (10)	○	○	○
Post-traumatic stress disorder (11)	○	○	○
Attention deficit hyperactivity disorder (ADHD) (12)	○	○	○
Eating disorder (13)	○	○	○
Autistic spectrum disorders (14)	○	○	○
Alcohol use disorder (15)	○	○	○

Substance use disorder (drugs) (16)	○	○	○
Suicidal thoughts (17)	○	○	○
Other (please describe) (18)	○	○	○

Page Break

Q5 If yes, please tick the mental health problems which a **father** you have worked with has experienced

	Yes (1)	No (2)	Don't know (3)
Depression (1)	○	○	○
Anxiety (2)	○	○	○
Panic attacks (3)	○	○	○
Psychosis (4)	○	○	○
Bipolar disorder (5)	○	○	○
Schizoaffective disorder (6)	○	○	○
Obsessive–compulsive disorder (7)	○	○	○
Post-traumatic stress disorder (8)	○	○	○
Attention deficit hyperactivity disorder (ADHD) (9)	○	○	○
Eating disorder (10)	○	○	○
Autistic spectrum disorders (11)	○	○	○
Alcohol use disorder (12)	○	○	○
Substance use disorder (drugs) (13)	○	○	○
Suicidal thoughts (14)	○	○	○
Other (please describe) (15)	○	○	○

Page Break

Q6 Have you encountered any of the following in families with pre-school children where a parent has a mental health problem?

	No (1)	Yes in the last month (2)	If yes, in the last year (3)	If yes, more than a year ago (4)
Socially isolated family (1)	○	○	○	○
Parents relationship broke down (2)	○	○	○	○
Parents cannot afford essentials for children (3)	○	○	○	○
Domestic abuse (4)	○	○	○	○
A child acts as carer for the parent (5)	○	○	○	○
A parent was imprisoned (6)	○	○	○	○
A child was identified as a 'child in need of care and support' (7)	○	○	○	○
A child was identified as 'at risk of harm' (8)	○	○	○	○
A child experienced attachment problems, if yes, please describe (9)	○	○	○	○
A child experienced developmental problems, if yes, please describe (10)	○	○	○	○
A child experienced behavioural	○	○	○	○

problems, if yes, please describe (11)				
Other, please describe (12)	○	○	○	○

Page Break

Q7 Do you use tools to assess maternal or paternal mental health?

- ○ Yes (1)
- ○ No, go to question 9 (2)

Q8 If yes, which of the following do you use? Please put in table the items as below with columns for mother and father.

	Have used with mother (1)	Have used with father (2)
FRAIT (Family Resilience Assessment Instrument and Tool) (1)	☐	☐
Whooley questions (2)	☐	☐
EPDS (Edinburgh Postnatal Depression Scale) (3)	☐	☐
Generalized Anxiety and Depression Scale (GADs) (4)	☐	☐
PHQ-9 (9)	☐	☐
An Attachment Assessment (5)	☐	☐
All Wales Maternity Record Questions (6)	☐	☐
A Local Assessment tool, please describe (7)	☐	☐
Other, please describe (8)	☐	☐

Page Break

Q9 Which of the following therapeutic strategies or interventions do you use when working with a mother with a mental health problem?

	Please tick all that apply (1)
I offer more one to one contacts (1)	○
I offer a package of listening visits/ non-directive counselling (2)	○
I use Mental Health First Aid- MHFA (12)	○
I offer facilitated self-help (23)	○
I encourage eligible mothers to attend Flying Start (3)	○
I invite her to attend a peer support group that I run or am involved with (4)	○
I refer to a peer support group run by another agency (5)	○
I refer to peer-led telephone support (22)	○
I refer to perinatal mental health services (6)	○
I refer to mental health services (7)	○
I refer to the GP (8)	○
I refer to family intervention services, such as family therapy (9)	○
I refer to voluntary agencies/ third sector services, please describe (10)	○
Other, please describe (11)	○

Page Break

Q10 Please indicate whether you agree or disagree with the following statements

	Strongly Agree (1)	Agree (2)	Neither agree nor disagree (3)	Disagree (4)	Strongly Disagree (5)
I am confident in supporting a mother who has mental health problem (1)	○	○	○	○	○
I am confident in supporting a father who has mental health problems (2)	○	○	○	○	○
My work with families where parents have a mental health problem helps to keep children safe (3)	○	○	○	○	○
I am often the only practitioner supporting parents with mental health problems because health visiting is a universal service (4)	○	○	○	○	○
There are clear guidelines for when to refer to mental health services (5)	○	○	○	○	○

Perinatal mental health services are easy to access in our area (6)	○	○	○	○	○
Third sector services contribute to keeping children safe when a parent has mental health problem (7)	○	○	○	○	○
Thresholds for children`s safeguarding services (social workers) are very high in my area (8)	○	○	○	○	○
Thresholds for mental health services are very high in my area (9)	○	○	○	○	○
There is a lack of support services available for people who do not meet thresholds for specialist mental health services (10)	○	○	○	○	○
We collect key performance data about the health visiting service we	○	○	○	○	○

offer which includes mental health (11)

Page Break

Q11 I feel confident about what action to take when a parent is experiencing a mental health problem

	Strongly Agree (1)	Agree (2)	Neither agree nor disagree (3)	Disagree (4)	Strongly disagree (5)
Please select (1)	○	○	○	○	○

Q12 I am confident in knowing which organisations to refer to when a parent has a mental health problem

	Strongly Agree (1)	Agree (2)	Neither agree nor disagree (3)	Disagree (4)	Strongly disagree (5)
Please select (1)	○	○	○	○	○

Q13 I have had training in supporting families when a parent has a mental health problem

	Yes, please select the training recieved (1)	No, go to question 14 (2)
As part of my HV training (1)	○	○
As part of my undergraduate nurse training (2)	○	○
As post qualification training (3)	○	○
As part of training to be part of an infant mental health team (4)	○	○
I am a qualified mental health nurse (5)	○	○
I have an infant mental health qualification (6)	○	○
I have Mental Health First Aid qualification (8)	○	○
Other (please describe) (7)	○	○

Page Break

Q14 Do you feel you have sufficient training to work effectively with families where a parent has a mental health problem?

If no, which of the following would be useful in your HV role? Please tick all that apply

	Useful (1)	Partly useful (2)	Not needed (3)
More training in keeping children safe when a parent has mental health problem (1)	○	○	○
More training on support available for parents in my local area (2)	○	○	○
More training in working with mothers with anxiety and mild to moderate depression (3)	○	○	○
More training in working with mothers with a mental health problem such as severe depression, bipolar disorder or personality disorder (4)	○	○	○
More training in working with fathers with mental health problems (5)	○	○	○
More training in recognising mental health problems in parents (6)	○	○	○
More interdisciplinary training on mental health problems (7)	○	○	○
More training in engaging the whole family, such as family therapy- type training (8)	○	○	○

Other, please describe (9)	○	○	○

Page Break

Q15 Please indicate whether you agree or disagree with the following statements the health visitors' role in relation to parental mental health issues

	Strongly Agree (1)	Agree (2)	Neither agree nor disagree (3)	Disagree (4)	Strongly disagree (5)
Supporting mothers with mental health problems is a key part of the health visitor's role (1)	○	○	○	○	○
Supporting fathers with mental health problems is a key part of the health visitor's role (2)	○	○	○	○	○
Health visitors can make a difference to family wellbeing when a parent has a mental health problem (3)	○	○	○	○	○
I do not currently have the time or capacity to work with mothers with mental health problems in my role as a health visitor (4)	○	○	○	○	○
I do not currently have the time or capacity to work with fathers with mental health problems in my	○	○	○	○	○

role as a health visitor (5)					
Health visitors refer clients to mental health services via the GP in the area where I work (6)	○	○	○	○	○
Health visitors are key members of the perinatal mental health team in the area where I work (7)	○	○	○	○	○
We work in collaboration with primary care to support parents with mental health problems (8)	○	○	○	○	○
We work in collaboration with outside agencies (e.g. third sector) to support parents with mental health problems (9)	○	○	○	○	○
The implementation of the Healthy Child Wales programme is helping health visitors keep children safe when a parent has a mental health problem (10)	○	○	○	○	○

The Welsh Government's new commitment to increasing the HV workforce will help keep children safe when a parent has a mental health problem (11)	○	○	○	○	○

Page Break

Q16 What are the most important ways health visitors can keep children safe when a parent has a mental health problem?
Please list the 5 most important interventions provided by HVs in order of priority

Click and drag in order of importance?

______ By knowing the family well (1)

______ By offering a universal service to all families (2)

______ By offering a targeted service when parents need more help (3)

______ By developing a relationship of trust with the family (4)

______ By focusing on the wellbeing and safety of the child (5)

______ By working well with mental health services (6)

______ By working well with the GP (7)

______ By working well with Flying Start and mainstream health visiting services (8)

______ By working well with children safeguarding agencies/social services children's safeguarding teams (9)

______ By working well with third sector agencies (10)

______ By being a key liaison person between all the agencies involved with the family (11)

Page Break

Q17 How can health visitors make the most difference when working with a family where a parent has a mental health problem?
Please describe

____

Q18 What are the barriers to health visitors effectively keeping children safe when a parent has a mental health problem?
Please describe

____

Q19 What helps you most as a health visitor to keep children safe effectively when a parent has a mental health problem?
Please describe

____

Q20 Please add any additional comments you would like to make in the space below.

____

Page Break

Thank you for completing this questionnaire. Your views will provide valuable information about how health visitors across Wales are working with pre-school children and their families.
Further information and a summary of the findings please contact Professor Louise Condon. L.J.Condon@swansea.ac.uk

Thank you for participating in the survey. If you would like to enter the prize draw to win £50, please email Mel Storey- m.storey@swansea.ac.uk

End of Block: Default Question Block
